# Investigate the relation of procalcitonin and prognosis in severe septic patients in SICU

**DOI:** 10.1186/2197-425X-3-S1-A870

**Published:** 2015-10-01

**Authors:** S Huang, Z Tang

**Affiliations:** The First Affiliated Hospital of Sun-Yat Sen University, SICU, Guangzhou, China; The First Affiliated Hospital of Sun-Yat Sen University, Guangzhou, China

## Introduction

In recent years, procalcitonin(PCT) ,as a new biological marker of systemic bacterial infection, is closely related to the prognosis of sepsis and its role in sepsis is becoming more and more attention.

## Objectives

To investigate the procalcitonin in relations with APCHE II score and prognosis in severe septic patients in SICU.

## Methods

To Collect 64 cases with severe sepsis hospitalized in SICU of the First Affiliated hospital of Sun Yat-sen University from December 2012 to December 2013, retrospectively. Monitored the dynamic changes of serum PCT,C-reactive protein(CRP), vital signs, blood routine test on the 1^st^, 3^rd^ and 7^th^ day after admission to SICU, the Acute Physiology and Chronic Health Evaluation II (APACHE II) scores were recorded. Compared the serum PCT, APACHE II, white blood cell(WBC), CRP and other inflammation markers between the two groups. The correlation of PCT and APACHE II was evaluated by spearman correlation analysis. The prediction of mortality of the PCT and APACHE II were evaluated by ROC curves.

## Results

There were 33 patients dead and 31 patients survived. The serum PCT level of the patients in the dead group were ((78.49 ± 193.77), (61.55 ± 176.70), (61.68 ± 197.89) ng/ ml) were significantly higher than that in the survived group ((10.70 ± 15.44),(10.60 ± 21.80), (5.67 ± 8.94)ng/ ml) on the 1^st^, 3^th^ and 7^th^ day(P < 0. 05).The APACHE II score of dead group (21.15 ± 6.93) on 1^st^ day was higher than that of survived group (18.06 ± 5.40) (p < 0.05). There is correlation between PCT and APACHEII .The area under the curve (AUC) was 0.625 (95%CI 0.49-0.76) for PCT, and 0.623 (95%CI 0.49-0.78) for APACHE II.

## Conclusions

Severe septic patient's PCT on 1^st^ day is valuable equal to APACHE II score in prognosis assessment.
